# *Haemonchus contortus*: siRNA mediated knockdown of matrix metalloproteinase 12A (MMP-12) results in reduction of infectivity

**DOI:** 10.1186/s13071-020-04025-1

**Published:** 2020-03-24

**Authors:** Muhammad Ali-ul-Husnain Naqvi, Hao Li, Wenxiang Gao, Sana Zahra Naqvi, Tahseen Jamil, Kalibixiati Aimulajiang, Lixin Xu, Xiaokai Song, Xiangrui Li, Ruofeng Yan

**Affiliations:** 1 MOE Joint International Research Laboratory of Animal Health and Food Safety, College of Veterinary Medicine, Nanjing Agricultural University, Nanjing, 210095 China; 2 Sindh Agriculture University, Tandojam, Sindh 70050 Pakistan

**Keywords:** Matrix metalloproteases 12A, RNA interference, siRNA, *Haemonchus contortus*, PBMCs

## Abstract

**Background:**

RNA interference (RNAi) is an important tool to determine the role of genes. RNAi has been widely used to downregulate target molecules, resulting in the reduction of mRNA for protein expression. Matrix metalloprotease 12A (MMP-12) is known to have important roles during embryonic development, organ morphogenesis and pathological processes in animals. However, MMP-12 from *Haemonchus contortus* has not been characterized.

**Methods:**

*Haemonchus contortus* MMP-12 gene was cloned and recombinant protein of MMP-12 (rHc-MMP-12) was expressed. Binding activities of rHc-MMP-12 to goat peripheral blood mononuclear cells (PBMCs) were assessed by immunofluorescence assay (IFA) and the immuno-regulatory effects of rHc-MMP-12 on cell proliferation and nitric oxide production were observed by co-incubation of rHc-MMP-12 with goat PBMCs. Furthermore, a soaking method was used to knockdown the expression of Hc-MMP12 gene using three siRNA, targeting different regions of the gene and infectivity of effective siRNA on the development of *H. contortus* was evaluated in goat.

**Results:**

rHc-MMP-12 was successfully expressed in an expression vector as well as the tissues of the cuticle of adult *H. contortus* worms and a successful binding with PBMCs surface were observed. Increased cellular proliferation and nitric oxide production by goat PBMCs was observed in a dose-dependent manner. Quantitative real time PCR (qRT-PCR) results confirmed the successful silencing of Hc-MMP-12 gene in siRNA of 1, 2 and 3 treated third-stage larvae (L3) of *H. contortus in vitro*. The most efficient qRT-PCR-identified siRNA template was siRNA-2, with a 69% suppression rate compared to the control groups. Moreover, in an *in vivo* study, silencing of the Hc-MMP-12 gene by siRNA-2 reduced the number of eggs (54.02%), hatchability (16.84%) and worm burden (51.47%) as compared to snRNA-treated control group. In addition, a shorter length of worms in siRNA-2-treated group was observed as compared to control groups.

**Conclusions:**

Our results indicate that siRNA-mediated silencing of Hc-MMP-12 gene in *H. contortus* significantly reduce the egg counts, larval hatchability, and adult worm counts and sizes. The findings of the present study demonstrate important roles of Hc-MMP-12 in the development of *H. contortus*.
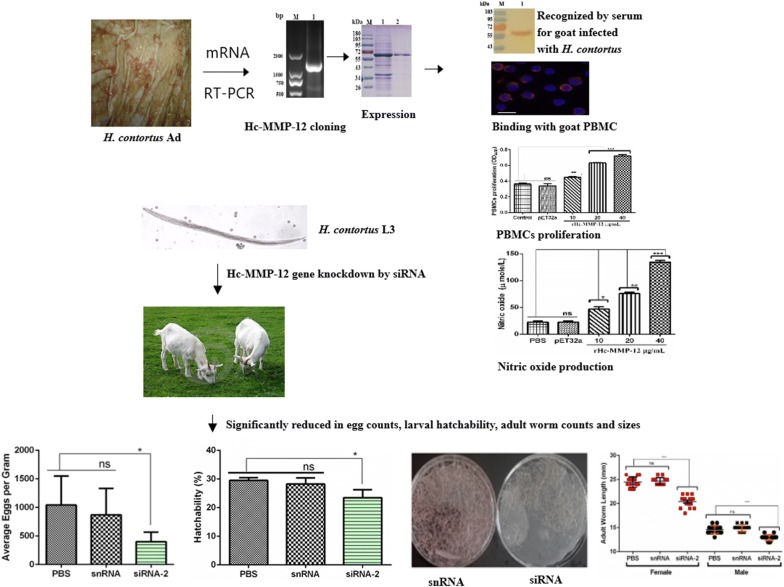

## Background

*Haemonchus contortus* is an important trichostrongylid nematode that affects small ruminants and causes significant economic losses due to high mortality and morbidity worldwide [1–3]. This nematode inhabits in the abomasum and has a complex life-cycle with a parasitic and a free-living phase [4]. Hosts become infected after the ingestion of third-stage infective larvae (L3). Blood-feeding adult *H. contortus* can lead to severe hemorrhagic gastritis, anemia, hypoproteinemia, edema, and even death in acute infections [5–7]. Control of this parasitic nematode is based on the application of anthelmintics, but excessive usage of these drugs has led to the development of anthelmintic resistance [8–11]. Previously, partial protection against *H. contortus* has been reported using DNA vaccines including actin [12], disorganized muscle family member-1 [13], cysteineprotease [14], glyceraldehyde-3-phosphate dehydrogenase [15] and glutathione peroxidase [16]. However, development of more effective control approaches is urgently needed.

Life-cycles of many nematode parasites consist of different migratory as well as invasive larval stages in the host environment [17]. Infective larvae (L3) of nematodes secrete macromolecules that are critical to infection and establishment of the parasite in the host [18]. In our previous study, comparative proteomics between *H. contortus* L3 and activated L3 was performed in which matrix metalloprotease 12A (MMP-12) was identified as one of the proteins that is upregulated during L3 exsheathment [19]. This protein belongs to a major group of zinc-dependent endopeptidases which have ability to cleave extracellular matrix constituents [18]. Moreover, matrix metalloproteases (MMPS) play important roles during embryonic development, organ morphogenesis and pathological processes [20, 21]. However, the exact biological functions of MMP-12 in developmental stages of *H. contortus* are not clear.

RNA interference (RNAi) is an important tool to determine the role of genes in which double-stranded RNA or small interfering (si)RNA activates the breakdown of homologous mRNA. RNAi has been widely used to downregulate target molecules, resulting in the reduction of mRNA for protein expression. Gene knockdown by RNAi was first described in a *Caenorhabditis elegans* model [22]. The success of RNAi in *C. elegans* can be attributed to the presence of the RNAi machinery, including the proteins with the ability to culture the nematode through generations. A gene knockdown technique has been practiced efficiently in parasites by the design and delivery of siRNA of target molecules. In previous studies, RNAi has been used to evaluate the biological functions of different genes in different helminths, for example paramyosin in *Trichinella spiralis* [23], type V collagen and calcium-regulated heat-stable protein in *Schistosoma japonicum* [24, 25], enolase in *Clonorchis sinensis* [26], and H11 aminopeptidase and DAF-3 in *H. contortus* [27, 28]. MMPS play important roles in developmental stages [20, 21] and the present study was designed to evaluate the biological roles of MMP-12 during different developmental stages of *H. contortus* in goat.

In this study, *H. contortus* MMP-12 was cloned, expressed in pET-32a, purified and confirmed through western blotting. Localization of MMP-12 in adult *H. contortus* worms and binding of recombinant MMP-12 with goat PBMCs were also performed. The modulatory effects of rHc-MMP12 on goat PBMCs proliferation and nitric oxide production were evaluated. Furthermore, a soaking method was used to knockdown the expression of the Hc-MMP12 gene using three siRNAs targeting different regions of the gene, followed by the evaluation of the efficiency on the development of *H. contortus*.

## Methods

### Animals and *H. contortus* infective third-stage larva (L3)

Local crossbred goats (*n* = 20), aged approximately 6 months, were bought from a farm in Xuyu county of Jiangsu Province and kept in the animal house of Nanjing Agricultural University under controlled conditions. All goats were administered with Levamisole orally (8 mg/kg body weight) twice at 2-week intervals to remove natural parasitic infections. Microscopical analysis of fecal samples was performed twice per week for helminth eggs. After 25 days of a second deworming, helminth-free goats were used in further experiments.

The *H. contortus* strain was maintained by serial passage in helminth-free goats at the Ministry of Education Laboratory at the Nanjing Agricultural University. To collect *H. contortus* infective L3, two local male goats (2-years-old) were raised under nematode-free conditions and dewormed twice at 15 days intervals using Levamisole. Both goats were infected using an oral dose of 10,000 L3 of *H. contortus*, and to confirm infection, fecal samples were collected and checked twice a week for the presence of *H. contortus* eggs. After confirmation of infection, infected larvae of *H. contortus* (L3) were obtained through conventional methods [29]. Briefly, feces from a *H. contortus* infected-goat were collected, crushed, mixed with water and combined with vermiculite to keep the mixture moist. The pan containing the mixture was covered with aluminum foil with several holes to allow air flow, and placed at room temperature for 10 days. To recover the larvae, the mixture was filtered through cheesecloth, larvae were examined microscopically, then preserved at 4 °C in water with 1% penicillin G until use.

### Molecular cloning of Hc-MMP-12

Total RNA was extracted from adult *H. contortus* using TRIzol reagent (Invitrogen, Shanghai, China) to clone the MMP-12 gene [30]. The cDNA was transcribed as per manufacturer’s instructions of the cDNA Kit used (Takara, Dalian, China) and stored at − 20 °C until use. Initially, the open reading frame (ORF) of Hc-MMP-12 was amplified by a reverse transcription-polymerase chain reaction (RT-PCR) using specific primers designed from the conserved domain sequences (CDS) of *H. contortus* (GenBank: HF964013.1). The following specific primers incorporating *BamH*I and *Xho*I digestion sites were used: forward primer (5′-CGC GGA TCC ATG ATA AGC GCC ACG CTG ATT CTG AT-3′) and reverse primer (5′- CCG CTC GAG TCA GAT GTA TTG GTA TTG AAA CGT G-3′). PCR reactions were performed in a total volume of 25 μl containing 12.5 μl 2× Taq master mix (Takara), 2 μl of cDNA, 8.5 μl ddH_2_O and 1 μl (50 pmol/µl) of each primer. PCR amplification was performed as follows: initial denaturation (one cycle) at 94 °C for 5 min, followed by 35 cycles at 94 °C for 30 s; 55 °C for 30 s; 72 °C for 2 min; and a final extension step at 72 °C for 10 min. EZNA Gel Extraction Kit (Omega Bio-tech, Norcross, GA, USA) was used according to the manufacturerʼs instructions to purify the PCR products, followed by ligation into the cloning vector pMD19-T (Takara, Dalian, China). Transformation of the recombinant plasmid (pMD19-T/MMP-12) into *Escherichia coli* DH_5α_ strain (Invitrogen Biotechnology, Shanghai, China) was performed and cultured in Luria Bertini (LB) medium containing ampicillin (100 µg/ml). The recombinant plasmid, pMD19-T/MMP-12, was identified by restriction enzyme digestion with *Bam*H I and *Xho*I. The MMP-12 gene was sub-cloned into pET-32a, followed by confirmation of successful insertion in the correct reading frame by sequencing (Invitrogen Biotechnology) and online BLAST analysis.

### Expression and purification of recombinant Hc-MMP-12

The recombinant plasmid (pET-32a ligated with Hc-MMP-12) was constructed, transformed into *E. coli* BL21 and cultured in LB containing ampicillin (100 μg/ml) at 37 °C. Protein was expressed by adding 1 mM isopropyl-β-d-thiogalactopyranoside (IPTG; Sigma-Aldrich, Shanghai, China). The recombinant Hc-MMP-12 protein was purified according to the manufacturer’s instructions using a Ni^2+^-nitrilotriacetic acid (Ni-NTA) column and refolded by renaturation buffer (20 mmol/l Tris-HCl, 500 mmol/l NaCl, 1 mmol/l GSH, 0.1 mmol/l GSSG, pH 8.0) containing different concentrations of urea (8, 6, 4, 2 and 0 M) [31]. The rHc-MMP-12 protein was detoxified using Toxin Eraser Endotoxin Removal Kit (GeneScript, Piscataway, USA).

### Preparation of polyclonal antibodies

Female Sprague Dawley (SD) rats of 150 g body weight (*n* = 6) were purchased from the Experimental Animal Center of Jiangsu, PR China (certified: SCXK 2008-0004). Rats were randomly divided into two groups, group 1 (*n* = 3) and group 2 (*n* = 3), kept in a sterilized room and supplied with food and water *ad libitum*.

The purified rHc-MMP-12 protein (0.3 mg in 0.5 ml PBS) was mixed with 0.5 ml of Freund’s complete adjuvant and injected subcutaneously to rats of group 1, while group 2 was kept untreated. Three more doses of protein (0.3 mg in 0.5 ml PBS) mixed with 0.5 ml of Freund’s incomplete adjuvant were injected with 2 weeks intervals. Sera samples from the control group and experimental group were collected 10 days after the last immunization and stored at − 80 °C until further use.

### Western blot assay

Western blotting played a preliminary role in the selection of the target protein to evaluate the immunogenicity and immuno-reactivity of the antigen [32]. Antigenic characteristics of rHc-MMP-12 were also evaluated in sera of goats infected with *H. contortus* as described previously [33]. First, purified rHc-MMP-12 was separated on 12% SDS-PAGE and a semi dry system (NovablotHoefer, San Francisco, USA) was used to transfer onto PVDF (polyvinyl difluoride membrane; Millipore, Billerica, USA) with transfer solution (Tris 48 mM, glycine 39 mM, SDS 0.0375%, methanol 20%). Blocking buffer (5% skimmed milk) diluted in TBS-T (Tris-buffered saline containing 0.05% Tween-20) was used to block the membrane at 37 °C for 2 h. The PVDF membrane was washed three times, cut into strips and incubated at 37 °C with 1:100 diluted primary antibody (anti-*H. cortortus* goat serum/anti-rHc-MMP-12 polyclonal antibody) for 2 h. After another three washes with TBS-T, the strips were incubated with 1:4000 diluted secondary antibody (Horseradish Peroxidase conjugated rabbit anti-goat IgG; Sigma-Aldrich, Hilden, Germany). Finally, immunoreactions were observed with the help of chromogenic substrate, 3-diaminobenzidine tetrahydrochloride (DAB; Tiangen Biotech, China).

### Localization of Hc-MMP-12

An immunofluorescence assay was performed according to the method stated previously [34]. Adult parasites were suspended in Tissue-Tek^®^ O.C.T. compound (Sakura, Torrance, CA, USA) after fixation on glass slides (poly-l-lysine hydrobromide) with 4% formaldehyde-0.2% glutaraldehyde in PBS for 45 min and snap frozen in liquid nitrogen. Worms were cut into pieces (10 µm thick) using a cryotome (CM1950; Frankfurt, Germany) and washed with PBS. The slides were treated with 5% BSA to block any non-specific binding followed by incubation with a 1:300 dilution of rat-anti-rHc-MMP-12 antiserum (experimental group) and normal rat serum (control group) as primary antibody at 37 °C. After 2 h incubation, slides of both groups were incubated in a 1:3000 dilution of Cy3-labeled goat anti-rat as second antibody (Beyotime, Shanghai, China) at 37 °C for 1 h. DAPI (diamidino-2-phenylindole) was used to stain the nuclei of worm cells and anti-fade fluoromount medium (Beyotime) was used before observing under confocal laser scanning microscope.

### Separation of PBMCs

PBMCs were separated from collected goat blood using the standard Ficoll-hypaque (GE Healthcare, Munich, USA) gradient centrifugation method as described previously [34]. After washing twice with Ca^2+^/Mg^2+^-free PBS (pH 7.4), PBMCs were adjusted to the required density (1 × 10^6^ cell/ml) in culture medium (RPMI 1640) containing 10% heat-inactivated fetal bovine serum (FBS), 100 U/ml penicillin or 100 mg/ml streptomycin (Gibco, Paisley, UK). Trypan blue exclusion test was performed to evaluate the cell viability as described previously [35].

### PBMCs binding assay

An immunofluorescence assay was performed to evaluate the binding ability of rHc-MMP-12 to goat PBMCs as described previously [30]. Briefly, freshly collected goat PBMCs were inoculated with 10 μg/ml rHc-MMP-12 and 10 μg/ml purified pET32a tag protein separately in a 24-well plate (1 ml/well). The plate was incubated at 37 °C in a humidified atmosphere with 5% CO_2_ for 1 h. Cells were washed in PBS and let to settle on poly-l-lysine coated glass slides for 20 min and fixed with 4% phosphate-buffered paraformaldehyde at room temperature for 30 min. The slides were blocked with PBS containing 5% BSA at 37 °C for 1 h. Subsequently, the slides were incubated with a 1:100 dilution of the primary antibody, rat anti-rHc-MMP-12 sera and normal sera (control slide) for 2 h. After three washes in PBS, slides were incubated in the dark with a secondary antibody, goat anti-rat IgG coupled with Cy3 (Beyotime; 1:1000 dilution) for 30 min. DAPI (Sigma-Aldrich) was subsequently added and the slides were incubated for 5 min at 37 °C in the dark. Finally, after washing slides were covered with a coverslip and immersed in anti-fade fluoromount solution (Beyotime). PBMCs were observed by confocal microscope with laser scanner (PerkinElmer, Waltham, MA, USA) at 100× magnification and digital images were taken using a Nikon microscope software package (Nikon, Tokyo, Japan).

### Cell proliferation assay

A cell proliferation assay was performed in triplicate using the cell counting kit-8 (CCK-8) assay reagent (Beyotime) as reported previously [36]. Briefly, freshly collected goat PBMCs (1 × 10^6^ cells/m) were seeded into 96-well plates and incubated with consecutive concentrations of rHc-MMP-12 (10, 20 and 40 μg/ml), concanavalin A (ConA), PBS and purified pET-32a tag protein (10 μg/ml) at 37 °C in a humidified atmosphere with 5% CO_2_ for 72 h. Before measuring the absorbance value (OD_450_) in micro plate reader (Thermo Fisher Scientific, Minneapolis, MN, USA), 10 μl of the CCK-8 reagent was added in each well for 4 h.

### Nitric oxide production assay

Goat PBMCs (1 × 10^6^ cells/ml) were washed twice with Ca^2+^/Mg^2+^-free PBS (pH 7.4) and 100 μl of cells were incubated respectively with serial concentrations of rHc-MMP-12 (10, 20 and 40 μg/ml), purified pET-32a tag protein and PBS in DMEM medium in a 96-well plate at 37 °C in a humidified atmosphere with 5% CO_2_ for 24 h. The nitric oxide (NO) production assay was performed in triplicate using a Griess assay as the described in the instructions of the Total Nitric Oxide Assay Kit (Beyotime). The absorbance values of the colored solution were measured using a microplate reader at 450 nm (OD_540_) and values converted to micro moles per liter (μmol/l) using a standard curve obtained by adding 0–80 μmol/l of sodium nitrate in fresh culture media. This experiment was performed three times individually.

### siRNAs knockdown of Hc-MMP-12

Three specific small interfering (si)RNAs were designed using the siRNA Design Tools program (https://rnaidesigner.thermofisher.com) based on the gene sequence of Hc-MMP-12, and chemically synthesized by Ribobio, Guangzhou, China (Additional file 1: Table S1). To knockdown Hc-MMP-12 gene expression, soaking method was used [25]. Initially, activated (sodium hypochlorite treated) *H. contortus* L3 were cultured in RPMI-1640 (supplemented with penicillin (500 units/ml) and streptomycin (500 mg/ml)) with siRNA-1, siRNA-2, siRNA-3, snRNA and PBS in 12-well culture plates (500 L3/500 μl) at 37 °C with 5% CO_2_. After 36 h incubation, 20% FBS was added. After another 24 h incubation, TRIzol reagent (Invitrogen, Shanghai, China) was used to extract total RNA from PBS, snRNA and siRNA treated L3 according to the manufacturer’s instructions. Subsequently, cDNA was synthesized as per the manufacturer’s instructions of the Takara cDNA kit and qRT-PCR was performed as described previously [34] to evaluate Hc-MMP-12 gene transcription. β-Actin was used as reference gene using specific primers (Additional file 1: Table S2). The most effective qRT-PCR identified siRNA (siRNA-2) was selected for further experiments.

### Infectivity of soaked siRNA-2 treated L3 *in vivo*

To evaluate the effects of soaked siRNA-2 treated L3 on production of *H. contortus* eggs and worms, 20 helminth-free goats were divided into four groups (*n* = 5 for each group). Group 1, Group 2 and Group 3 were infected orally with 8000 activated L3 treated with PBS, snRNA (RNAi control) and siRNA-2, respectively. Group 4 was kept untreated as a control. Fecal samples were collected from all groups at 7, 14, 21, 23, 25, 27, 29, 31 and 35 days post-infection (dpi). McMaster egg count was performed for fecal samples as descripted previously [37]. Additionally, the hatchability percentage of eggs was estimated at 29, 31 and 33 dpi. Finally, at 35 dpi the goats of groups treated with soaked siRNA-2, PBS and snRNA were humanely euthanized, the abomasum immediately opened and the presence and length of adult worms were recorded.

## Results

### Molecular cloning, sequence analysis and western blotting of Hc-MMP-12

**Fig. 1:**
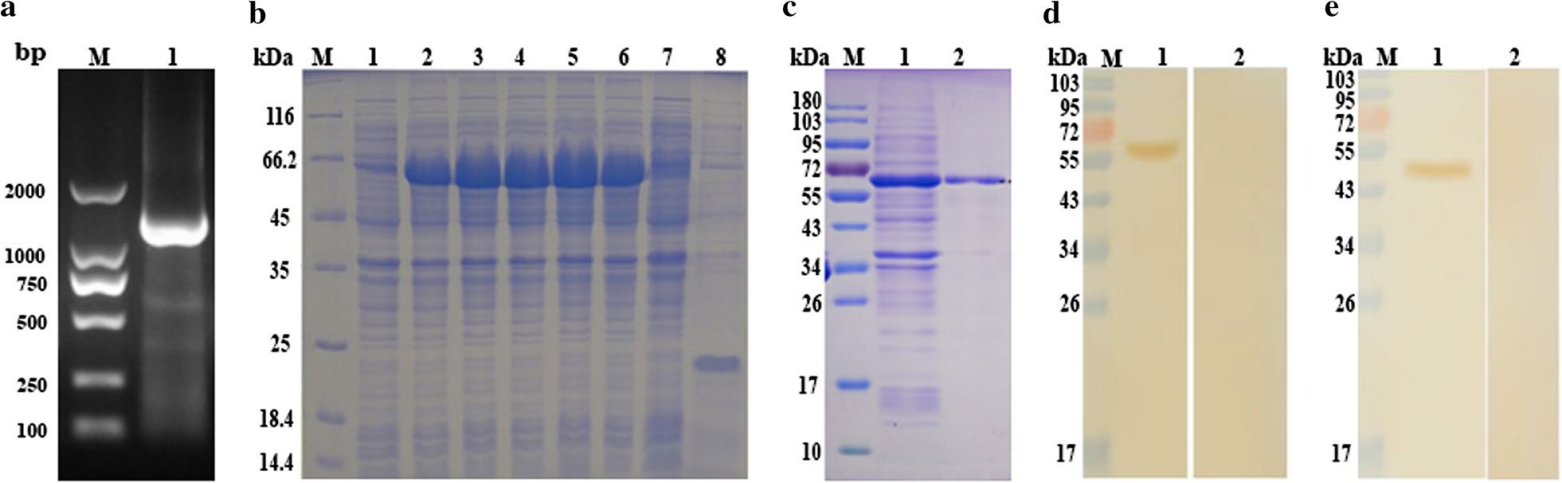
Cloning, expression, purification and western blot analysis of Hc-MMP-12. **a** Agarose gel electrophoresis of the PCR products. Lane M: DNA molecular marker; Lane 1: the PCR generated fragment of MMP-12 gene with molecular size of 1275 bp. **b** SDS-PAGE. Lane M: standard molecular weight protein marker; Lane 1: expression before IPTG induction; Lanes 2–6: expression after IPTG induction at different time points; Lane 7: pET32a protein before purification; Lane 8: purified pET32a protein. **c** Purification of recombinant MMP-12. Lane 1: recombinant MMP-12 protein before purification; Lane 2: recombinant MMP-12 protein after purification. **d** Western blot analysis of rHc-MMP-12. Lane 1: serum sample from *H. contortus* experimentally infected goat; Lane 2: serum sample from normal goat. **e** Western blot analysis of naïve protein. Lane 1: serum sample from SD rats containing polyclonal antibodies against rHc-MMP-12; Lane 2: serum sample from normal SD rats

The amplified PCR products of the Hc-MMP-12 gene were obtained from cDNA of *H. contortus* using a specific pair of primers and a correct fragment size of 1275 bp encoding a protein of 424 amino acids was detected (Fig. 1a). The recombinant plasmid of Hc-MMP-12 (pET-32a/Hc-MMP-12) induced with IPTG and expressed in *Escherichia coli* BL21 cells showed a His6-tagged Hc-MMP-12 protein on SDS-PAGE after Coomassie blue staining (Fig. 1b) that was purified with a denaturation procedure followed by Ni^2+^ affinity chromatography and a single band was detected with a calculated molecular weight of *c.*64 kDa (Fig. 1c). Moreover, western blot analysis showed that rHc-MMP-12 protein could be recognized by serum from a goat experimentally infected with *H. contortus* (Fig. 1d, Lane 1) and naive protein could be recognized by anti*-*Hc-MMP-12 polyclonal antibodies (Fig. 1e, Lane 1). However, no protein was detected with normal goat or rat sera (Fig. 1d, e; Lane 2). The results indicated that Hc-MMP-12 had good antigenicity and could be recognized by the immune system of the host.

### Localization of Hc-MMP-12 in adult male/female *H. contortus* worms

**Fig. 2:**
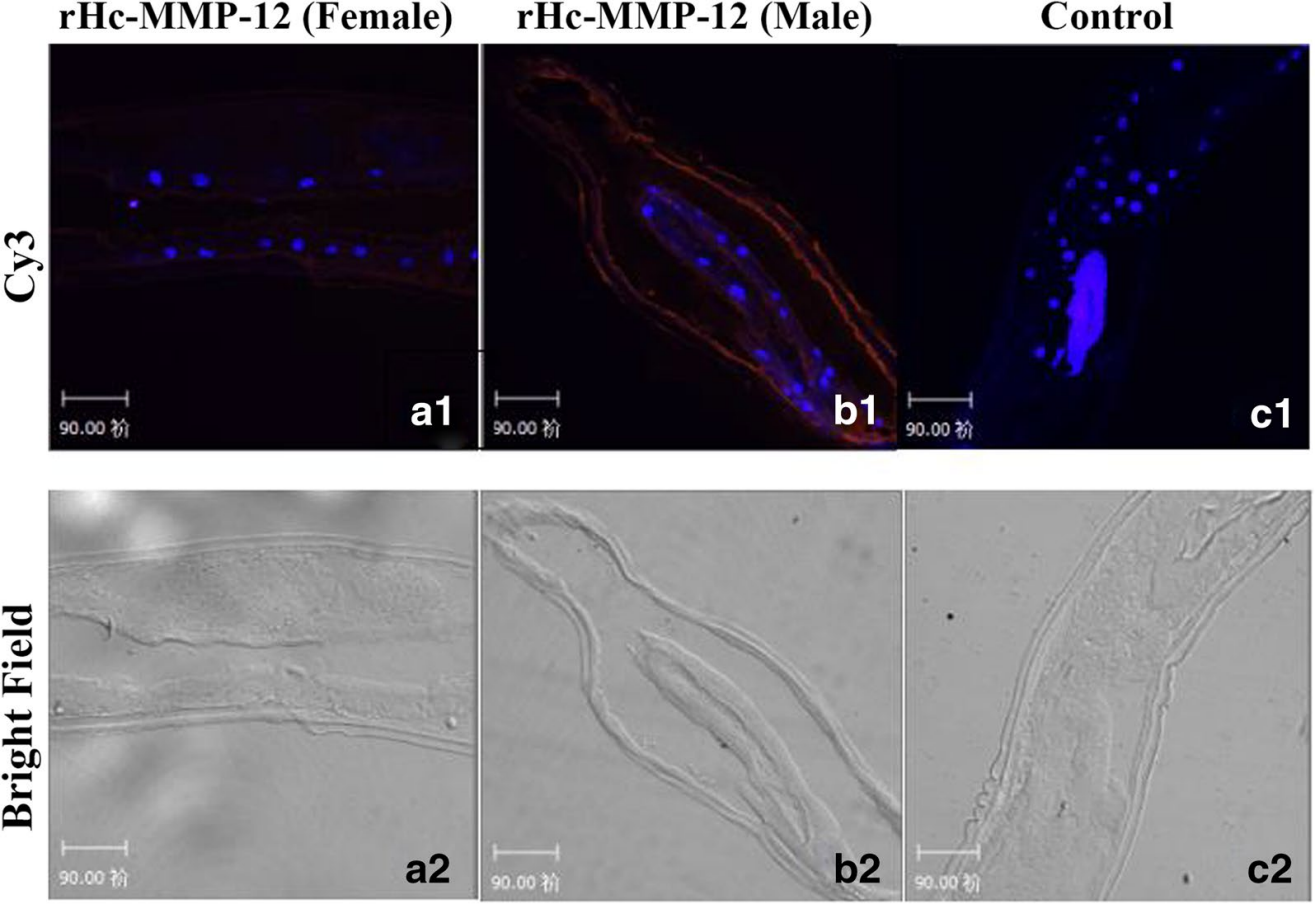
Localization of Hc-MMP-12 protein in adult *H. contortus* worm by immunofluorescence assay. Nuclei were stained with DAPI (blue) and target protein with Cy3 (red). **a1** Targeted Hc-MMP-12 protein localized in the tissues of female worms. **b1** Targeted Hc-MMP-12 protein localized in the tissues of male worms. **c1** No fluorescence was observed in control. **a2**, **b2**, **c2** Micrographs with bright field. *Scale-bars*: 90 μm

An immunofluorescence assay was performed using partial body sections of *H. contortus* male and female worms to localize the Hc-MMP-12. Nuclei were stained with DAPI (blue) and target protein with Cy3 (red). The results indicated that Hc-MMP-12 was prominent in muscles of adult female and male nematodes (Fig. 2) and might be localized on the outer and inner surfaces of the cuticle. No protein was observed in the control group.

### Effects of rHc-MMP-12 on different functions of goat PBMCs

**Fig. 3:**
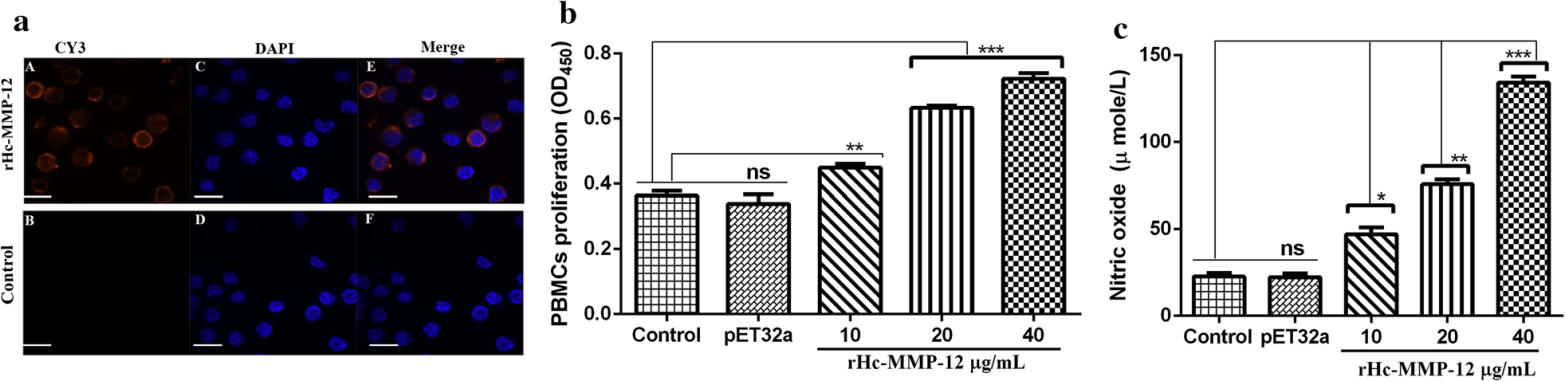
Influence of rHc-MMP-12 on different functions of goat peripheral blood mononuclear cells (PBMCs). **a** Binding of rHc-MMP-12 with PBMCs. PBMCs were cultured with rHc-MMP-12 (10 μg/m) and without protein as control at 37 °C and 5% CO_2_. After 2 h incubation of PBMCs with rHc-MMP-12, rat anti-rHc-MMP-12, or negative rat IgG (as first antibody) was added, followed by staining with Cy3-conjugated secondary antibody and DAPI. Red fluorescence on surface of cells shows target protein staining (Cy3) and cell nuclei were visualized by DAPI (blue). No red fluorescence was observed in control groups. **b** Influence of rHc-MMP-12 on PBMCs proliferation. Cells were incubated with different concentrations of rHc-MMP-12, purified pET32a protein and PBS (control) at 37 °C and 5% CO_2_ for 72 h. Cell proliferation index was calculated considering the optical density (OD 450) values in PBS control as 100%. **c** Influence of rHc-MMP-12 on nitric oxide production by PBMCs *in vitro*. Cells were incubated with PBS, pET32a and serial concentrations of rHc-MMP-12 protein for 24 h in 37 °C and 5% CO_2_. The nitrate concentration in the PBMCs was measured by using the Griess assay. Statistically significant differences between the means of three independent experiments were assessed using a one-way ANOVA (*P < 0.05, **P < 0.01, ***P < 0.001; ns, no significant difference)

An immunofluorescence assay was performed to confirm the binding of rHc-MMP-12 with goat PBMCs. Confocal microscope imaging revealed that rHc-MMP-12 protein could bind on the surface of goat PBMCs. As indicated in Fig. 3a, nuclei and cells were stained with blue fluorescence and target protein with red, whereas there was no fluorescence observed in the control section. Moreover, the effects of rHc-MMP-12 on cell proliferation were measured by the cell counting kit (CCK-8). Results highlighted that proliferation was significantly increased (ANOVA: *F*_(4, 25)_ = 95.98, *P* < 0.001) by the PBMCs incubated with 10 μg/ml, 20 μg/ml and 40 μg/ml concentration of rHc-MMP-12 as compared to the control group. No significant difference was observed between the control and purified pET32a protein groups (Fig. 3b). Furthermore, the total nitric oxide assay kit used to evaluate the effect of rHc-MMP-12 on nitric oxide production by PBMCs revealed that goat PBMCs incubated with 10 μg/ml, 20 μg/ml and 40 μg/ml concentration of rHc-MMP-12 showed a significant increase (ANOVA: *F*_(4, 10)_ = 242.50, *P* < 0.001) in NO production as compared to the control group. However, no significant difference in NO production was observed between PBS (control) and purified pET32a protein groups (Fig. 3c).

### Transcriptional level of Hc-MMP-12 in siRNA-treated L3 *in vitro*

**Fig. 4:**
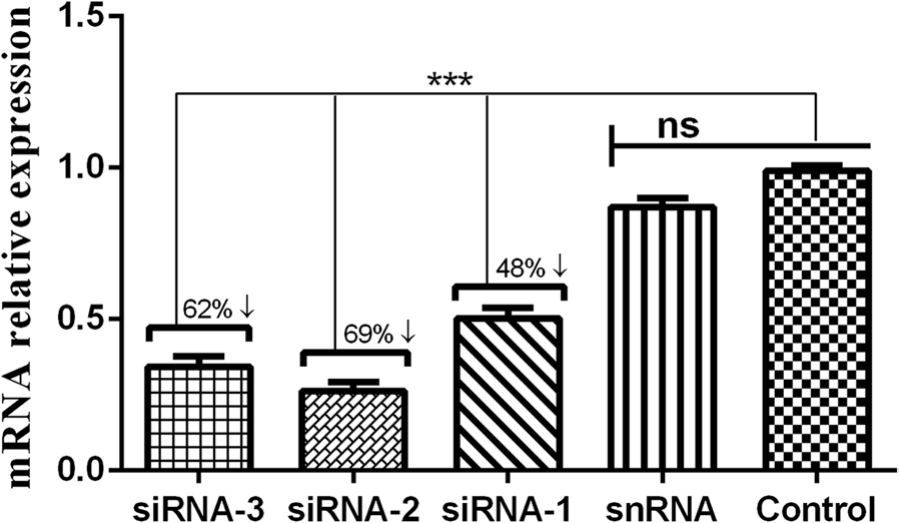
Transcription level of Hc-MMP-12 in soaked siRNA-treated *Haemonchus contortus* L3. Infective *H. contortus* L3 were treated with siRNA-1, siRNA-2, siRNA-3, negative control siRNA (snRNA) and PBS. Statistically significant differences between the means of three independent experiments were assessed using a one-way ANOVA (***P < 0.001; ns, no significant difference)

Infective *H. contortus* L3 were treated with siRNA-1, siRNA-2, siRNA-3, control siRNA and PBS for 60 h to knockdown Hc-MMP-12 gene expression using the soaking method. Transcriptional levels of Hc-MMP-12 in siRNA-treated L3 were evaluated using qRT-PCR to assess whether Hc-MMP-12 was successfully silenced. qRT-PCR analysis revealed a significant reduction in Hc-MMP-12 transcript levels in L3 larvae treated with Hc-MMP-12 siRNA-1 (48 ± 0.79%), siRNA-2 (69 ± 0.57%), and siRNA-3 (62 ± 1.04%) compared with snRNA template and PBS control (ANOVA: *F*_(4, 10)_ = 118.30, *P* < 0.001). The most significant suppression in transcription level of Hc-MMP-12 mRNA was observed in siRNA-2-treated L3 (Fig. 4). There was no significant difference in transcription level observed between the snRNA and the PBS-treated L3 group. The most efficient qRT-PCR-identified siRNA (siRNA-2) which was selected for further experiments.

### Effects of Hc-MMP-12-knockdown on egg production and hatchability *in vivo*

**Fig. 5:**
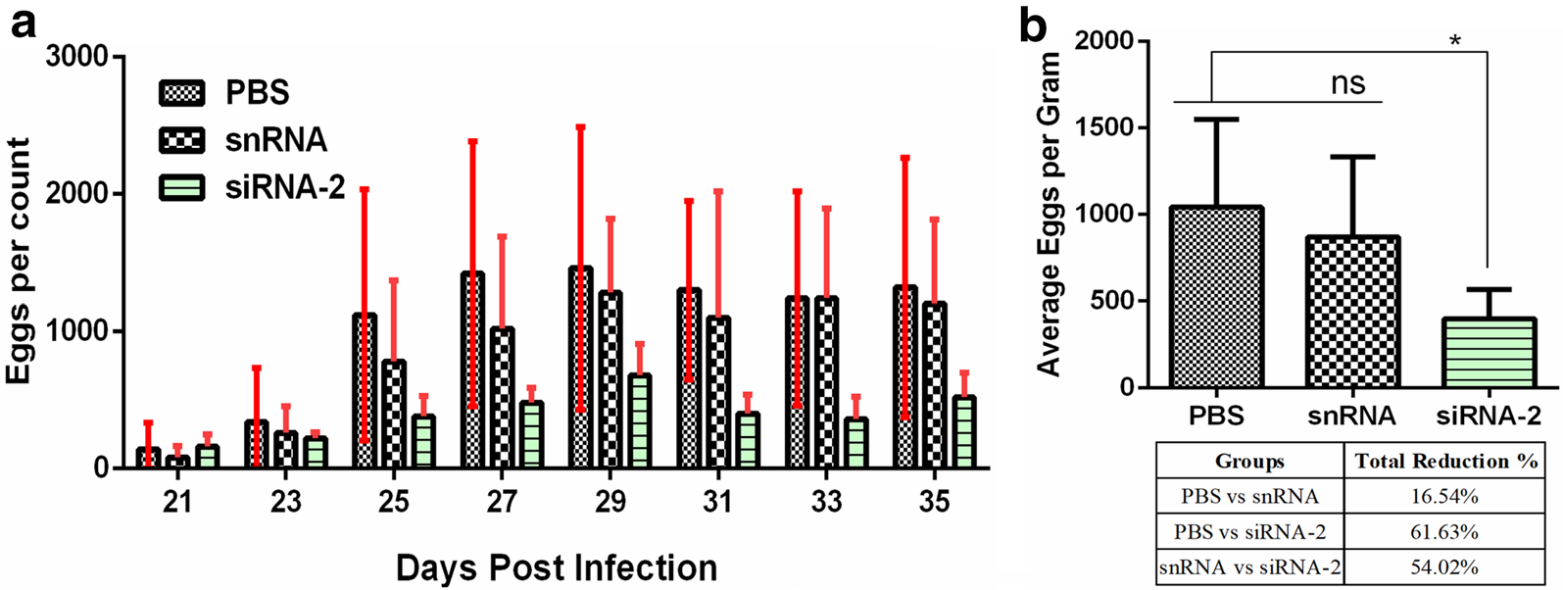
Effect of siRNA mediated knockdown of Hc-MMP-12 on eggs per gram feces. Fecal samples were collected from goats infected with L3 treated with siRNA-2, snRNA and PBS. **a** Eggs per gram at different days post-infection. **b** Total egg count of different groups. Significant reduction in average EPG was observed in the siRNA-2-treated group compared to PBS group

**Fig. 6:**
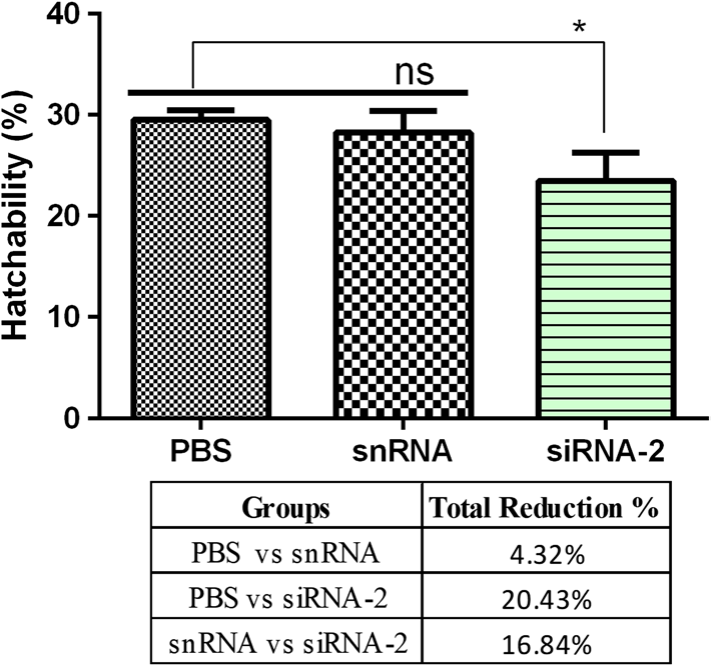
Effect of siRNA-mediated knockdown of Hc-MMP-12 on the total hatchability rate of different groups. Statistically significant differences were assessed using a one-way ANOVA (*P < 0.05; ns, no significant difference)

Suppression of Hc-MMP-12 mRNA in *H. contortus* was performed by soaked siRNA-2. To evaluate the effects of soaked siRNA-2-treated L3 on *H. contortus* egg production, the McMaster technique was performed using fecal samples collected at different dpi from goats infected with soaked siRNA-2, PBS and snRNA-treated L3. Goats infected with soaked siRNA-2-treated L3 showed a significant reduction in the number of eggs per gram as compared to goats infected with PBS- and snRNA-treated L3 (ANOVA: *F*_(2, 21)_ = 5.31, *P* = 0.014; Fig. 5). No eggs were detected in fecal samples of all groups collected at 7 and 14 dpi and also of the control groups. Additionally, reduced hatchability rate of eggs collected from the soaked siRNA-2 treated group was observed compared with PBS- and snRNA-treated groups (ANOVA: *F*_(2, 6)_ = 6.86, *P* = 0.028; Fig. 6). Moreover, no significant difference in eggs per gram and hatchability rate was observed between PBS-treated and snRNA-treated groups.

### Infectivity of Hc-MMP-12-knock down on worm burden and morphology *in vivo*

**Fig. 7:**
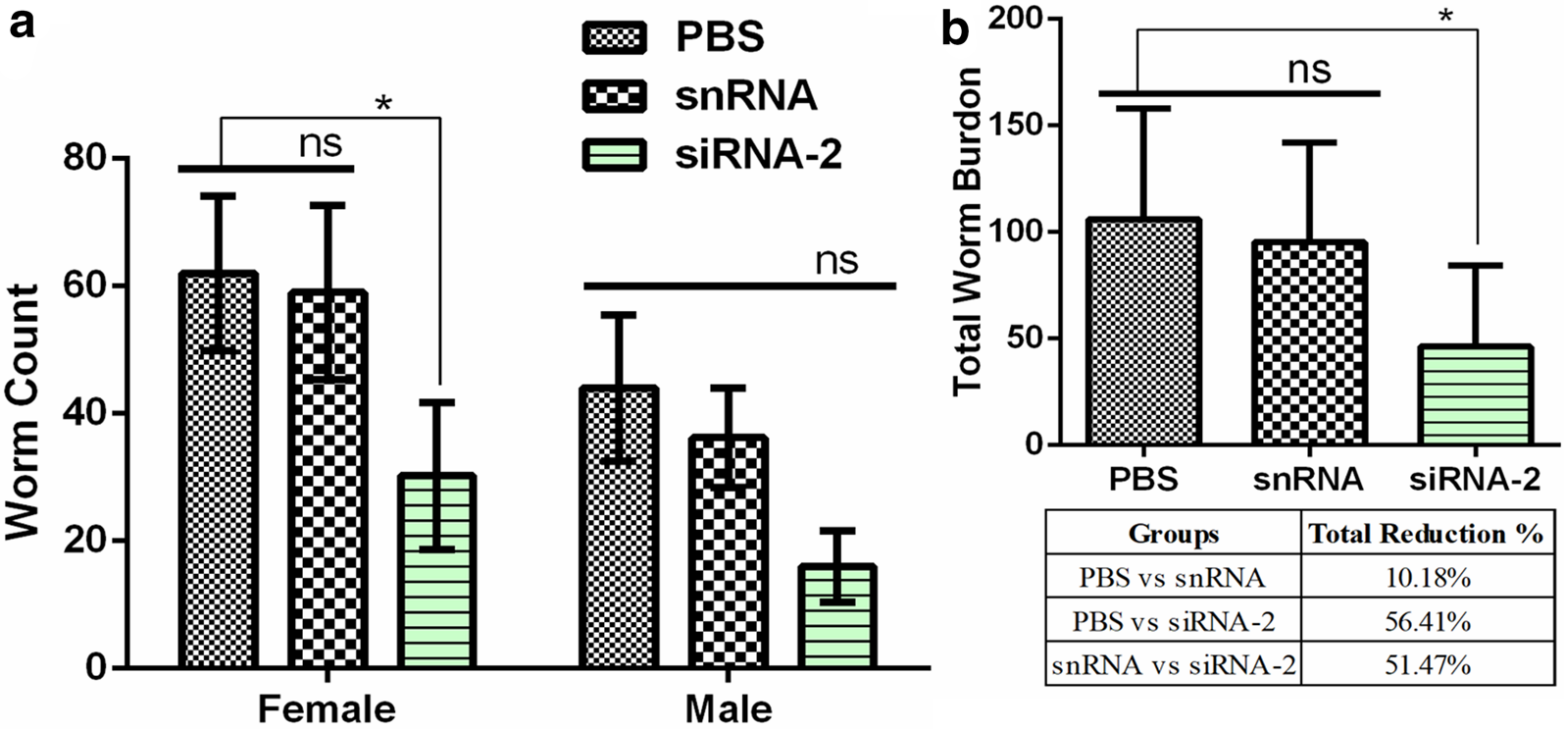
Effect of siRNA mediated knockdown of Hc-MMP-12 on adult *H. contortus* worm burden. **a** Male and female worm count. **b** Total worm count. Goats were examined at 35 days post-infection and adult worms were collected and counted. Statistically significant differences were assessed using a one-way ANOVA (*P < 0.05; ns, no significant difference)

**Fig. 8:**
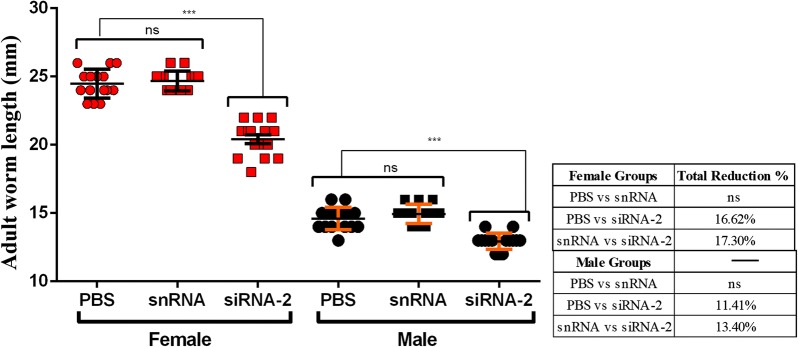
Effect of siRNA mediated knockdown of Hc-MMP-12 on the length of adult *H. contortus* worms. Goats were examined at 35 days post-infection and adult worms were collected, and length was measured. Statistically significant differences were assessed using a one-way ANOVA (***P < 0.001; ns, no significant difference)

Necropsy was performed to evaluate the effects of Hc-MMP12 on worm burden. Worm count results showed that female worms were significantly reduced in goats infected with soaked siRNA-2 treated L3 compared to PBS-treated and snRNA-treated goats (ANOVA: *F*_(2, 12)_ = 6.61, *P* = 0.012). Moreover, the number of male worms collected from the siRNA-2-treated group was non-significant compared to the number of male worms collected from PBS- and snRNA-treated groups. Ordinary siRNA-2 treated goats showed 56.41% and 51.47% reduction rate in total worm count when compared with PBS- and snRNA-treated groups, respectively (Fig. 7). In addition, female worms in the Hc-MMP-12 siRNA-2-treated group were smaller compared with the PBS- and snRNA-treated control groups (body length: ANOVA: *F*_(2, 42)_ = 81.76, *P* < 0.001), and there was no difference in worm length between the two control groups. Similarly, the length of male worms in the Hc-MMP-12 siRNA-2-treated group as compared to PBS- and snRNA-treated control groups was shorter (ANOVA:*F*_(2, 42)_ = 33.70, *P* < 0.001), and there was no difference in worm length between the two control groups (Fig. 8).

## Discussion

Gastrointestinal parasitic infection is one of the major health problems in the world that causes huge economic loss in livestock production [38]. Several broad-spectrum anthelmintics are used to control these parasitic infections, but anthelminthic resistance of nematodes in small ruminants is becoming a serious threat in some countries [8–11]. Therefore, development of more effective control approaches is urgently needed.

ESPs are molecules secreted *in vivo* or *in vitro* by the worms and are involved in complex functions of host parasite interactions [29]. In the present study, MMP-12 was detected with *H. contortus* infected goat sera that confirmed it as one of the *H. contortus* ESPs. In addition, rHc-MMP-12 was also detected by anti-Hc-MMP-12 rat sera when native protein was loaded in SDS-PAGE. In our previous proteomic analysis, MMP-12 was found at adult and late adult developmental stages of *H. contortus in vivo* [29]. In the present study, MMP-12 was localized immunohistochemically in both female and male adult *H. contortus* worms. These results indicated that MMP-12 is one of the excretory and secretory products of *H. contortus* that may play an important role during *H. contortus* development. In previous studies, immuno-regulatory roles of ESPs have been reported in different cellular processes such as cell adhesion and cell proliferation [39]. Cell proliferation is very important for the development of immune responses and tissue regeneration. Cell proliferation is regulated by both T cells and antigen-presenting cells (APCs) [40] and increases the number of immune effector cells. In the present study, it was identified that goat PBMC proliferation increased at a significant level which ultimately showed constant apoptosis of PBMC in response to rHc-GDC treatment. The proliferation results of the present study are in line with the results of the previous study [5]. In contrast, cell proliferation was suppressed after PBMCs were treated with rHcARF1 [41]. Specific immuno-regulatory properties of antigen might be the possible explanation for these differences.

Nitric oxide (NO) has been documented as an important immunomodulator in various infections including *H. contortus* by mediating host protection, parasite killing or suppressing their growth. Previously, it was documented that NO had been involved in a non-specific defense mechanism against *H. contortus* and various other parasites [42]. In the present study, immunomodulatory effects of the rHc-GDC on NO production by goat PBMCs were evaluated. Cells incubated with different concentrations of MMP-12 significantly increased the NO production in a dose-dependent manner. A constant increase in NO production indicated that MMP-12 was involved in the immunomodulatory regulation of NO on goat PBMCs. A previous study supports these results in which one ESP, recombinant Galectin domain containing protein, was used [34]. Insights into the mechanisms of NO production are still lacking.

RNAi technology is an interesting method and has been widely used as an important method to study gene function in molecular biology. It is also intensively applied in studying parasite gene function identification, including nematodes [23, 24, 43]. In parasitic nematodes, this technique was successfully performed in *Nippostrongylus brasiliensis* [44]. In the present study, infective *H. contortus* L3 were treated with siRNA-1, siRNA-2, siRNA-3, control siRNA and PBS to knockdown Hc-MMP-12 gene expression by using the soaking method *in vitro*. Transcriptional levels of Hc-MMP-12 in siRNA-treated L3 were evaluated using qRT-PCR to assess whether Hc-MMP-12 was successfully silenced. qRT-PCR analysis confirmed the successful knockdown of the MMP-12 gene in *H. contortus* L3 *in vitro*. Previously, successful silencing of the DAF-3 gene by siRNA in *H. contortus* was reported which is in line with findings of the present study [28]. To evaluate the effects of Hc-MMP-12 siRNA-2 *in vivo*, a further experiment was performed whereby goats were infected with soaked siRNA-2-treated L3, snRNA-treated L3 and PBS-treated L3. Effects of soaked siRNA-2-treated L3 on *H. contortus* egg production, eggs hatchability at different dpi and on worm burden were evaluated. Results revealed that goats infected with siRNA-2-treated L3 showed a significant reduction in egg production, hatchability and worm burden when compared to goats infected with PBS-treated L3. Furthermore, the length of *H. contortus* adult worms collected from siRNA-2-treated group was shorter when compared to the worms from snRNA-treated groups. These findings indicate that silencing of Hc-MMP-12 gene by siRNA has the potential to reduce infectivity of *H. contortus*. Collectively, these results provide evidence that Hc-MMP-12 may play a role in the development of *H. contortus* and will help to further understand the host-parasite interaction.

## Conclusions

This study revealed that rHc-MMP-12 is an important active protein of *H. contortus* ESPs that may play a crucial role in the immune regulation and developmental stages of the parasite. The results indicated that the interaction of rHc-MMP-12 with host cells increased the production of nitric oxide and cell proliferation in a dose-dependent manner. siRNA-mediated knockdown of Hc-MMP-12 also significantly reduced egg burden and hatchability, and adult worm burden and size.

## Supplementary information

**Additional file 1: Table S1.** Specific small interfering RNAs of the MMP-12 gene. **Table S2.** Primer sequences of target genes related to quantitative real-time PCR.

## Data Availability

All data generated or analyzed during this study are included in this published article and its additional file.

## Abbreviations

ESPs: excretory-secretory products
MMP-12: matrix metalloproteases 12A
RNAi: RNA interference
SI: small interfering
ORF: open reading frame
RT-PCR: reverse transcription-polymerase chain reaction
LB: Luria Bertini
IPTG: isopropyl-β-d-thiogalactopyranoside
Ni-NTA: Ni^2+^-nitrilotriacetic acid
PVDF: polyvinyl difluoride
TBS-T: tris-buffered saline containing 0.05% Tween-20
DAB: 3-diaminobenzidine tetrahydrocholoride
CCK-8: cell counting kit-8
NO: nitric oxide

## References

[1] Tariq KA, Chishti MZ, Ahmad F, Shawl AS (2008). Epidemiology of gastrointestinal nematodes of sheep managed under traditional husbandry system in Kashmir valley. Vet Parasitol.

[2] Nwosu CO, Madu PP, Richards WS (2007). Prevalence and seasonal changes in the population of gastrointestinal nematodes of small ruminants in the semi-arid zone of north-eastern Nigeria. Vet Parasitol.

[3] Naqvi MA, Naqvi SZ, Memon MA, Aimulajiang K, Haseeb M, Xu L (2019). Combined use of indirect ELISA and western blotting with recombinant hepatocellular carcinoma-associated antigen 59 is a potential immunodiagnostic tool for the detection of prepatent *Haemonchus contortus* infection in goat. Animals.

[4] Dey AR, Zhang Z, Begum N, Alim MA, Hu M, Alam MZ (2019). Genetic diversity patterns of *Haemonchus contortus* isolated from sheep and goats in Bangladesh. Infect Genet Evol.

[5] Wang QQ, Wu LY, Hasan MW, Lu MM, Wang WJ, Yan RF (2019). Hepatocellular carcinoma-associated antigen 59 of *Haemonchus contortus* modulates the functions of PBMCs and the differentiation and maturation of monocyte-derived dendritic cells of goats *in vitro*. Parasites Vectors.

[6] Soulsby EJL (1982). Helminths, arthropods and protozoa of domesticated animals.

[7] Schallig HDFH, Hornok S, Cornelissen JBWJ (1995). Comparison of two enzyme immunoassays for the detection of *Haemonchus contortus* infections in sheep. Vet Parasitol.

[8] Han T, Wang M, Zhang G, Han D, Li X, Liu G (2017). Gastrointestinal nematodes infections and anthelmintic resistance in grazing sheep in the Eastern Inner Mongolia in China. Acta Parasitol.

[9] Zhang Z, Gasser RB, Yang X, Yin F, Zhao G, Bao M (2016). Two benzimidazole resistance-associated SNPs in the isotype-1 β-tubulin gene predominate in *Haemonchus contortus* populations from eight regions in China. Int J Parasitol Drugs Drug Resist.

[10] Kaplan RM (2004). Drug resistance in nematodes of veterinary importance: a status report. Trends Parasitol.

[11] Wang C, Li F, Zhang Z, Yang X, Ahmad AA, Li X (2017). Recent research progress in China on *Haemonchus contortus*. Front Microbiol.

[12] Yan R, Wang J, Xu L, Song X, Li X (2014). DNA vaccine encoding *Haemonchus contortus* Actin induces partial protection in goats. Acta Parasitol.

[13] Yan R, Sun W, Song X, Xu L, Li X (2013). Vaccination of goats with DNA vaccine encoding Dim-1 induced partial protection against *Haemonchus contortus*: a preliminary experimental study. Res Vet Sci.

[14] Muleke CI, Ruofeng Y, Yanming S, Osuga IM, Shivairo RS, Xiangrui L (2013). Cellular immune response and abomasum worm burden in goats vaccinated with HC58cDNA vaccine against *H. contortus* infection. Adv Life Sci Technol.

[15] Han K, Xu L, Yan R, Song X, Li X (2012). Vaccination of goats with glyceraldehyde-3-phosphate dehydrogenase DNA vaccine induced partial protection against *Haemonchus contortus*. Vet Immunol Immunopathol.

[16] Han K, Xu L, Yan R, Song X, Li X (2012). Molecular cloning, expression and characterization of enolase from adult *Haemonchus contortus*. Res Vet Sci.

[17] Lai SC, Jiang ST, Chen KM, Lee HH (2005). Matrix metalloproteinases activity demonstrated in the infective stage of the nematodes, *Angiostrongylus cantonensis*. Parasitol Res.

[18] Williamson AL, Lustigman S, Oksov Y, Deumic V, Plieskatt J, Mendez S (2006). *Ancylostoma caninum* MTP-1, an astacin-like metalloprotease secreted by infective hookworm larvae, is involved in tissue migration. Infect Immun.

[19] Wang F, Xu L, Song X, Li X, Yan R (2016). Identification of differentially expressed proteins between free-living and activated third-stage larvae of *Haemonchus contortus*. Vet Parasitol.

[20] Stetler-stevenson WG, Aznavoorian S, Liotta A (1993). Tumor cell interactions with the extracellular matrix during invasion and metastasis. Annu Rev Cell Biol.

[21] Sato H, Seiki M (1996). Membrane-type matrix metalloproteinases (MT-MMPs) in tumor metastasis. J Biochem.

[22] Fire A, Xu S, Montgomery MK, Kostas SA, Driver SE, Mello CC (1998). Potent and specific genetic interference by double-stranded RNA in *Caenorhabditis elegans*. Nature.

[23] Chen X, Yang Y, Yang J, Zhang Z, Zhu X (2012). RNAi-mediated silencing of paramyosin expression in *Trichinella spiralis* results in impaired viability of the parasite. PLoS ONE.

[24] Yang Y, Jin Y, Liu P, Shi Y, Cao Y, Liu J (2012). RNAi silencing of type V collagen in *Schistosoma japonicum* affects parasite morphology, spawning, and hatching. Parasitol Res.

[25] Zou X, Jin YM, Liu PP, Wu QJ, Liu JM, Lin JJ (2011). RNAi silencing of calcium-regulated heat-stable protein of 24 kDa in *Schistosoma japonicum* affects parasite growth. Parasitol Res.

[26] Wang Li CJ, Hu DD, Liu RD, Wang Z (2014). Identification of early diagnostic antigens from major excretory-secretory proteins of *Trichinella spiralis* muscle larvae using immunoproteomics. Trop Biomed.

[27] Samarasinghe B, Knox DP, Britton C (2011). Factors affecting susceptibility to RNA interference in *Haemonchus contortus* and *in vivo* silencing of an H11 aminopeptidase gene. Int J Parasitol.

[28] Di W, Liu L, Zhang T, Li F, He L, Wang C (2019). A DAF-3 co-Smad molecule functions in *Haemonchus contortus* development. Parasites Vectors.

[29] Gadahi JA, Wang S, Bo G, Ehsan M, Yan R, Song X (2016). Proteomic analysis of the excretory and secretory proteins of *Haemonchus contortus* (HcESP) binding to goat PBMCs *in vivo* revealed stage-specific binding profiles. PLoS ONE.

[30] Gadahi JA, Li B, Ehsan M, Wang S, Zhang Z, Wang Y (2016). Recombinant *Haemonchus contortus* 24 kDa excretory/secretory protein (rHcES-24) modulate the immune functions of goat PBMCs *in vitro*. Oncotarget.

[31] Zhang ZC, Huang JW, Li MH, Sui YX, Wang S, Liu LR (2014). Identification and molecular characterization of microneme 5 of *Eimeria acervulina*. PLoS ONE.

[32] El-Hassan EM, El-Bahr SM (2013). Antigenic and immunogenic components of *Haemonchus longistipes* identified by western immunobloting. Am J Biochem Biotechnol.

[33] Naqvi MAH, Jamil T, Naqvi SZ, Memon MA, Aimulajiang K, Aleem MT (2020). Immunodiagnostic potential of recombinant tropomyosin during prepatent *Haemonchus contortus* infection in goat. Res Vet Sci.

[34] Naqvi MA, Memon MA, Jamil T, Naqvi SZ, Aimulajiang K, Gadahi JA (2020). Galectin domain containing protein from *Haemonchus contortus* modulates the immune functions of goat PBMCs and regulates CD4+ T-helper cells *in vitro*. Biomolecules.

[35] Strober W (1997). Trypan blue exclusion test of cell viability. Curr Protoc Immunol.

[36] Yuan C, Zhang H, Wang W, Li Y, Yan R, Xu L (2015). Transmembrane protein 63A is a partner protein of *Haemonchus contortus* galectin in the regulation of goat peripheral blood mononuclear cells. Parasites Vectors.

[37] Ljungström S, Melville L, Skuce PJ, Höglund J (2018). Comparison of four diagnostic methods for detection and relative quantification of *Haemonchus contortus* eggs in feces samples. Front Vet Sci.

[38] Sori T, Dhuguma R, Kiros Y (2006). Epidemiology of gastrointestinal parasites of ruminants in western Oromia, Ethiopia. Intern J Appl Res Vet Med.

[39] Gadahi JA, Yongqian B, Ehsan M, Zhang ZC, Wang S, Yan RF (2016). *Haemonchus contortus* excretory and secretory proteins (HcESPs) suppress functions of goat PBMCs *in vitro*. Oncotarget.

[40] Loke P, MacDonald AS, Robb A, Maizels RM, Allen JE (2000). Alternatively activated macrophages induced by nematode infection inhibit proliferation *via* cell-to-cell contact. Eur J Immunol.

[41] Gadahi JA, Ehsan M, Wang S, Zhang Z, Yan R, Song X (2017). Recombinant protein of *Haemonchus contortus* small GTPase ADP-ribosylation factor 1 (HcARF1) modulate the cell mediated immune response *in vitro*. Oncotarget.

[42] James SL (1995). Role of nitric oxide in parasitic infections. Microbiol Rev.

[43] Wang ZQ, Zhang SB, Jiang P, Liu RD, Long SR, Zhang X (2015). The siRNA-mediated silencing of *Trichinella spiralis* nudix hydrolase results in reduction of larval infectivity. Parasitol Res.

[44] Hussein AS, Kichenin K, Selkirk ME (2002). Suppression of secreted acetylcholinesterase expression in *Nippostrongylus brasiliensis* by RNA interference. Mol Biochem Parasitol.

