# Supplementary material for: Haemonchus contortus: siRNA mediated knockdown of matrix metalloproteinase 12A (MMP-12) results in reduction of infectivity
Source: Parasit Vectors. 2020 Mar 24;13:151. doi: 10.1186/s13071-020-04025-1 (PMC7092576; doi:10.1186/s13071-020-04025-1)
Supplement: Supplementary file 1 — Additional file 1: Table S1. Specific small interfering RNAs of the MMP-12 gene. Table S2. Primer sequences of target genes related to quantitative real-time PCR. [file 13071_2020_4025_MOESM1_ESM.docx]

**Additional file 1**

**Table S1. Specific small interfering RNAs of MMP-12 gene**

| **MMP-12** | **Forward 5 → 3** | **Reverse 5 → 3** | **Position** |
| --- | --- | --- | --- |
| siRNA-1 | GCUCUUCGUCCAGAGGAAUTT | AUUCCUCUGGACGAAGAGCTT | 222-241 |
| siRNA-2 | CCGAGAGAUUGCUCAGUAUTT | AUACUGAGCAAUCUCUCGGTT | 494-513 |
| siRNA-3 | GCCAAAGGUUCUGAAAGAUTT | AUCUUUCAGAACCUUUGGCTT | 770-789 |
| snRNA | GCACUUCUCCUAGAGUCCATT | UUGCAAGCGAGAGUAUGCGTT | -- |

**Table S2. Primer sequences for Quantitative real-time PCR**

| Gene Name | Forward 5 → 3 | Reverse 5 → 3 | Amplification size (bp) | *Amplification efficiency (%) |
| --- | --- | --- | --- | --- |
| MMP-12 | CAACTTCTCAGAGTGCGATTTG | TTGAGTGGAGCCTTTATCCAGT | 205 | 95.12 |
| Beta Tubulin | TGCATTGGTACACTGGAGAAG | GCTTCCTGGTACTGCTGATATT | 240 | 91.23 |

^*^Amplification efficiency (%) = (10^-1/slope^ -1) ×100
